# Beauty in the beast – Placozoan biodiversity explored through molluscan predator genomics

**DOI:** 10.1002/ece3.11220

**Published:** 2024-04-11

**Authors:** Michael Eitel, Hans‐Jürgen Osigus, Bastian Brenzinger, Gert Wörheide

**Affiliations:** ^1^ GeoBio‐Center Ludwig‐Maximilians‐Universität München München Deutschland; ^2^ Department of Earth and Environmental Sciences, Paleontology and Geobiology Ludwig‐Maximilians‐Universität‐München München Deutschland; ^3^ Institut für Tierökologie Stiftung Tierärztliche Hochschule Hannover Hannover Deutschland; ^4^ Staatliche Naturwissenschaftliche Sammlungen Bayerns (SNSB) – Zoologische Staatssammlung München Deutschland; ^5^ Staatliche Naturwissenschaftliche Sammlungen Bayerns (SNSB) – Bayerische Staatssammlung für Paläontologie und Geologie München Deutschland; ^6^ Present address: Hochschulbibliothek, Stiftung Tierärztliche Hochschule Hannover Hannover Deutschland

**Keywords:** ecological niche, interstitium, placozoan biodiversity, placozoan predators, Rhodopidae

## Abstract

The marine animal phylum Placozoa is characterized by a poorly explored cryptic biodiversity combined with very limited knowledge of their ecology. While placozoans are typically found as part of the epibenthos of coastal waters, known placozoan predators, namely small, shell‐less sea slugs belonging to the family Rhodopidae (Mollusca: Gastropoda: Heterobranchia), inhabit the interstitium of seafloor sediment. In order to gain further insights into this predator–prey relationship and to expand our understanding of placozoan ecological niches, we screened publicly available whole‐body metagenomic data from two rhodopid specimens collected from coastal sediments. Our analysis not only revealed the signatures of three previously unknown placozoan lineages in these sea slug samples but also enabled the assembly of three complete and two partial mitochondrial chromosomes belonging to four previously described placozoan genera, substantially extending the picture of placozoan biodiversity. Our findings further refine the molecular phylogeny of the Placozoa, corroborate the recently established taxonomic ranks in this phylum, and provide molecular support that known placozoan clades should be referred to as genera. We finally discuss the main finding of our study – the presence of placozoans in the sea floor sediment interstitium – in the context of their ecological, biological, and natural history implications.

## Graphical abstract

Previous assumptions about the primary habitat of Placozoa are challenged, and their broader diversity beyond disc‐shaped grazers in coastal waters is revealed. The discovery of placozoan‐predatory sea slugs prompts consideration of a new ecological niche: seafloor sediment interstices. Metagenomic analysis of these sea slugs confirms the presence of placozoan DNA, expanding our understanding of their biodiversity and ecological significance.

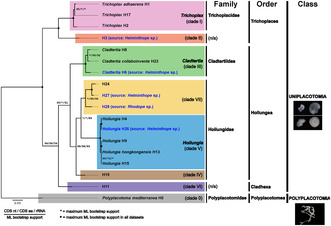

## 1 INTRODUCTION

Placozoans are flat, millimeter‐sized, and disk‐shaped marine benthic animals. They are found in shallow marine environments, often living on the surfaces of benthic substrates such as rocks, corals, shells, mangrove roots, and other submerged material (Eitel et al., 2013; Eitel & Schierwater, 2010; Grell & Benwitz, 1971; Pearse & Voigt, 2010; Schierwater, 2005; Signorovitch et al., 2006; Voigt et al., 2004). Here they feed primarily on algae and organic matter that they digest extracellularly to take up particles by pinocytosis (Eitel et al., 2013; Grell & Ruthmann, 1991). Rarely have they been described as preying on other animals, such as stolons of colonial Hydrozoa (Pearse & Voigt, 2010). One of the most unique features of placozoans is their simple body plan. They lack a nervous system, digestive system, or specialized organs, instead relying on a diffuse network of cells to perform basic physiological functions (Grell & Benwitz, 1971, 1981; Smith et al., 2014). Placozoans reproduce asexually by binary fission and budding, where a new individual grows from the parent organism (Thiemann, 1990; Thiemann & Ruthmann, 1988, 1991). They can also reproduce sexually, although the details of this process are not well understood (Eitel et al., 2011; Grell, 1971, 1972; Grell & Benwitz, 1974).

Placozoans are regularly found in tropical and subtropical waters, but they have also been found in temperate waters, such as the Mediterranean, the northern Atlantic Ocean, or the Southern Indian Ocean off Southern Australia (Eitel et al., 2013; Eitel & Schierwater, 2010; Signorovitch et al., 2006). A phylogeographic study has indicated that longitudinal dispersal patterns might be correlated with individual molecular clades (Eitel & Schierwater, 2010). Because of their small size and cryptic nature, placozoans are difficult to study in the wild. However, laboratory studies have provided some insights into their ecology. For example, it has been found that placozoans are able to survive and divide under a wide range of environmental conditions, including temperature, salinity, and food source, while showing sensitivity to ocean global warming and acidification (Schleicherová et al., 2017). They have also been observed to exhibit some degree of behavioral plasticity, such as movement toward light and chemical attractants (Heyland et al., 2014).

To date, four placozoan species have been accepted: *Trichoplax adhaerens* Schulze (1883), *Hoilungia hongkongensis* Eitel, Schierwater & Wörheide (2018), *Polyplacotoma mediterranea* Osigus & Schierwater (2019), and *Cladtertia collaboinventa* Tessler, Neumann, Osigus, DeSalle & Schierwater (2022), with multiple molecular genetic lineages awaiting formal description. Recent phylogenomics research led to the establishment of a taxonomy of the Placozoa, including the erection of higher taxonomic ranks (Tessler et al., 2022). Some genetic lineages still lack genomic data and holotype specimens, making it challenging to assign them taxonomically. Therefore, the previously established haplotype and clade system (Eitel et al., 2013; Eitel & Schierwater, 2010; Grell & Benwitz, 1971; Pearse & Voigt, 2010; Schierwater, 2005; Signorovitch et al., 2006; Voigt et al., 2004) is still provisionally applied to undescribed placozoans, pending the acquisition of holotype material, despite its incremental numbering system not reflecting natural relationships. The taxonomic classification of the Placozoa, however, remains essential for comprehending their evolutionary history and ecology, offering a more nuanced understanding of the diversity within these simple yet fascinating organisms. Different placozoan taxa may have distinct ecological preferences, such as living in different types of habitats or using different food sources. Mapping the placozoan taxonomic classification onto a geographic map will help to identify generalists that adapt to various environmental conditions and specialists that require specific habitats and their unique features (cf. Eitel & Schierwater, 2010).

The small placozoans are typically collected using a benthos sampling approach that relies on chance encounters (see Eitel et al., 2013 for details). This method involves placing clean microscopic slides into seawater at various marine habitats, allowing a natural biofilm to form over time, and screening the resulting slides for placozoans under a light microscope. Although an improved sample processing method involving alcohol treatment of field samples has been developed (Miyazawa & Nakano, 2018), visually identifying placozoans on natural substrates such as stones or mussel shells remains challenging and time‐consuming, with a high risk of missing specimens due to their small size and transparent appearance. Therefore, new sampling strategies must be developed to better explore the poorly understood diversity of placozoans.

In this study, we propose a novel “catch‐by‐slug” placozoan detection approach to investigate placozoan diversity in the field. This strategy takes advantage of the feeding behavior of Rhodopidae sea slugs, microscopic epibenthic or interstitial gastropods from worldwide tropical to subtropical regions, which actively prey on placozoans (Haszprunar & Heß, 2005; Jörger et al., 2020). *Rhodope veranii* Koelliker, 1847 and *Rhodope placozophagus* Cuervo‐González, 2017, for instance, can exclusively live, grow, and reproduce on placozoan prey (Cuervo‐González, 2017; Riedl, 1959). *Helminthope psammobionta* Salvini‐Plawen, 1991, a particularly thin‐bodied animal living in deeper regions of the sediment, has not been observed feeding, but anatomy suggests that the feeding style is essentially the same as in *Rhodope* species (Brenzinger et al., 2013; Jörger et al., 2020). These sea slugs ingest their placozoan prey completely (smaller individuals) or in pieces by a specialized cilia‐mediated suction; digestion then takes place in the tubular digestive gland (Brenzinger et al., 2011), where undigested material may persist in the central duct between digestive cells for some time, providing an opportunity to indirectly detect placozoan DNA in the sea slug's gut. To achieve this, we leveraged existing metagenomic data from a range of animal phyla (Sevigny et al., 2021), which included epibenthic to interstitial *Rhodope* and fully interstitial *Helminthope* sea slugs. Using the publicly available sequencing data from these sea slugs, we were able to detect an unexpected placozoan diversity and even reconstruct the entire mitochondrial chromosomes of several placozoans from two randomly collected sea slug individuals. This approach has allowed us to gain new insights into the diversity and distribution of placozoans in marine sediments, an ecosystem that has been excluded as a suitable habitat for placozoans in previous research (Pearse & Voigt, 2010; Signorovitch et al., 2006).

## 2 METHODS

### 2.1 Placozoan mitochondrial metagenome assembly and annotation

To investigate the potential presence of placozoan mitochondrial DNA, we utilized Illumina whole animal genomic paired‐read sequencing data from two hitherto undescribed Rhodopidae species, *Rhodope* sp. and *Helminthope* sp., which were publicly available (Sevigny et al., 2021). Two datasets were available for each species, one enriched for mitochondrial DNA and one non‐enriched metagenomic DNA pool, and both datasets were obtained from a single whole individual, including gut material. The samples were isolated by Sevigny et al. (2021) from homogeneous marine sediment (coarse sand) collected underwater near the shore of Isla Iguana in the Pacific Ocean, Panama.

To process reads and perform SPAdes assemblies, short wrapper bash scripts were set up for both sea slug species. The scripts as well as the initial metagenomic assemblies are available in a GitHub repository at https://github.com/PalMuc/beauty_in_the_beast. The reads were first trimmed using Trimmomatic v0.39 (Bolger et al., 2014) to remove adaptors, followed by error correction using Karect v1.0 (Allam et al., 2015). The final assembly step was performed using a stand‐alone installation of SPAdes v3.14.1 (Prjibelski et al., 2020) with the ‘meta’ option. This option was chosen to prevent the generation of chimeric contigs potentially originating from similarities between mollusc and placozoan mitochondrial DNA sequences and/or further prey contamination.

After assembly, all publicly available placozoan mitochondrial genomes (for details and accessions, see Miyazawa et al., 2021) were blasted individually against the SPAdes contigs in Geneious Prime 2019.2.3 (Kearse et al., 2012; https://www.geneious.com) using the discontinuous megablast algorithm and an evalue of 1e−10.

Candidate placozoan mitochondrial contigs were identified and lengthened in Geneious for 10 iterations using all error‐corrected read pairs. The extensions were performed independently for the *Rhodope*‐ and *Helminthope*‐derived placozoan raw contigs, respectively. Extended placozoan raw contigs of the *Helminthope* sample were then mapped to the complete mitochondrial DNA sequence of the placozoan taxa H3, H24, *Hoilungia* H4, and *Cladtertia* H8. These sequences were used as references since the alignment of 16S‐encoding metaSPAdes contigs with the commonly used placozoan 16S barcoding fragment indicated the presence of closely related *Hoilungia* haplotypes in the *Helminthope* sp. dataset. Placozoan mitochondrial DNA contigs of the *Rhodope* sp. dataset were mapped to H24 since a related haplotype was found in the metagenome assembly according to the 16S barcoding fragment.

We merged extended contigs at overlapping sites, as indicated by the reference mitochondrial sequence. In the case of sequence gaps, the reference sequence covering the gap region (plus 300 bp on both sides of the gap) was blasted against all trimmed reads in Geneious using the discontinuous megablast algorithm and an evalue of 1e−10. The resulting blast hits were paired and assembled with metaSPAdes implemented in Geneious using 33, 55, and 77 nt as kmers, respectively. The newly assembled contigs, together with the initially extended contigs, were again mapped to the respective reference mitochondrial DNA, and overlaps were merged again. The final remaining gaps were filled with “N” according to the number of missing nucleotides in the reference mtDNA. All resulting mitochondrial scaffolds and contigs were manually checked for assembly artifacts by mapping the trimmed reads to the final placozoan scaffolds/contigs. The error‐corrected reads were not used for this step to see if error correction might have been flawed due to the presence of multiple different placozoan haplotypes in the *Helminthope* sp. DNA pool.

The placozoan mitochondrial genomes were manually annotated in Geneious by aligning the new sequences with mitochondrial sequences of all haplotypes within the same or closely related clade, using the MAFFT E‐INS‐i algorithm with default settings (Katoh & Standley, 2013). After alignment, annotations were transferred, and mitochondrial protein‐coding sequences (CDS) were confirmed by identifying open reading frames (ORFs) using the ‘Mold, Protozoan, and Coelenterate mitochondrial’ genetic code “4.” The tRNA and ribosomal DNA sequences were annotated based on their sequence similarity to reference placozoan mitochondrial DNAs.

Additionally, we used BLAST (setting as above) to screen the metagenomic sequence data for placozoan ribosomal DNA, utilizing the 18S and 28S sequence data available for placozoans in GenBank. Furthermore, we searched for signatures of mitochondrial DNA and ribosomal DNA from other prey species. These searches were conducted using blastn (setting as above), employing the sequences of the identified host mitochondrial chromosomes/contigs and the nuclear ribosomal DNA (18S + 5.8S + 28S) unit. For contig IDs utilized in the BLAST searches, please refer to Table S1.

### 2.2 Phylogenetic inferences

To perform phylogenetic analyses, protein‐coding nucleotide sequences were extracted from both new and previously available placozoan mitochondrial DNAs. Coding sequences (CDS) for individual genes were aligned using the MAFFT E‐INS‐i algorithm and the ‘Translation Align’ option in Geneious. The ‘CDS nt’ data matrix was obtained by concatenating all coding sequence alignments. A ‘CDS aa’ matrix was also created by translating the nucleotide sequences using the ‘Mold, Protozoan, and Coelenterate mitochondrial’ genetic code “4” and aligning the resulting protein sequences using the MAFFT L‐INS‐i algorithm. The ‘rDNA’ matrix was generated by concatenating alignments of the mitochondrial ribosomal DNA sequences, including both mitochondrial ribosomal DNAs (12S and 16S). Alignments of the combined 16S fragments and the 12S sequences, respectively, were performed independently with default settings using the MUSCLE (Edgar, 2004) algorithm, implemented in Geneious.

All three data matrices were analyzed using Maximum Likelihood (ML) in IQ‐TREE multicore v2.0.3 (Minh et al., 2020; Nguyen et al., 2015) and Bayesian inferences (BI) carried out in MrBayes v3.2.7a (Ronquist et al., 2012). The best‐fitting substitution models according to the AICc were determined using modeltest‐ng (Darriba et al., 2020) and used in the inferences. ML analyses included 1000 bootstrap replicates with *Polyplacotoma mediterranea* (herein haplotype H0 (Osigus et al., 2019)) set as the outgroup. Bayesian inferences were run until convergence. Our extracted sequences of placozoan haplotype H3 were excluded from phylogenetic inferences due to high incompleteness, and instead, the publicly available complete mitochondrial genome sequence of this haplotype (Signorovitch et al., 2007) was used. All input and output files of all phylogenetic analyses are available online in the public repository.

### 2.3 Distance calculations

In MEGA v.11.0.13 (Tamura et al., 2021), mean pairwise genetic distances were calculated based on the amino acid alignments. The following settings were used: model/method = p‐distance; gaps/missing = pairwise. All taxa were assigned to groups, and mean distances were calculated for orders within classes, families within orders, genera within families, species within genera, and haplotypes within clades. To evaluate the major split in the Placozoa, calculations were performed both between Poly‐ and Uniplacotomia taxa and within Uniplacotomia alone. For each gene, the average of all mean group distances was generated, and the values were compared between taxonomic ranks.

## 3 RESULTS

### 3.1 New placozoan taxa in predatory sea slug genomic data

**FIGURE 1:**
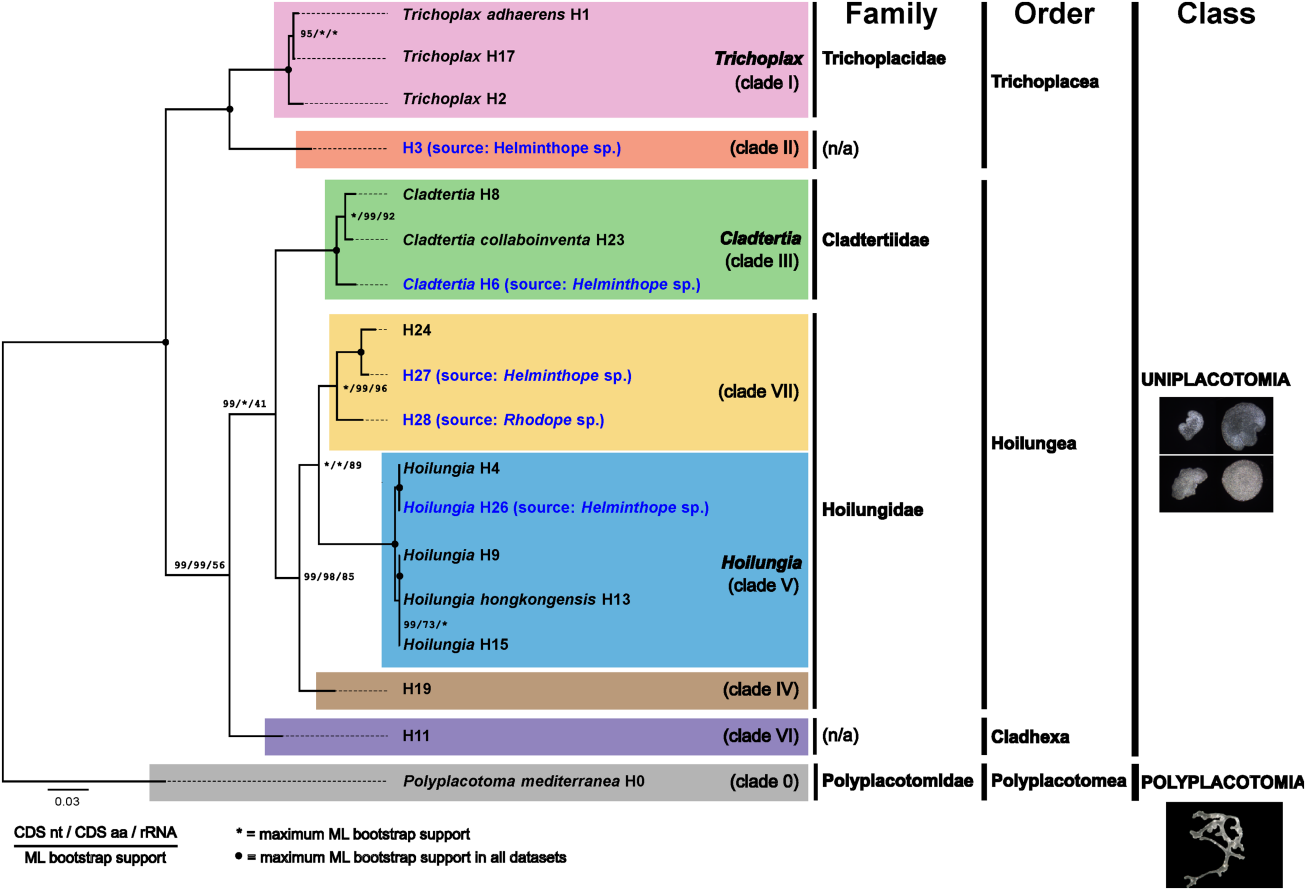
Phylogenetic relationships of 18 placozoans based on complete mitogenome data. The shown phylogram is based on the Bayesian inference of concatenated alignments of the nucleotide sequence of 12 protein‐coding genes and was rooted in *Polyplacotoma mediterranea*. The grouping of placozoan clades is identical to previous analyses (Miyazawa et al., 2021). Species/haplotypes in black font are based on previously published mitochondrial DNA sequences, while blue font highlights the discovered lineages, three of which are new to science. In the *Helminthope* metagenomic dataset, two previously known placozoans were identified: haplotypes H3 and H6. While for H3, the mitochondrial genome was previously published (Signorovitch et al., 2006), for H6, only the 16S barcoding fragment was known. The complete mitochondrial sequence confirms the 16S‐based grouping of sp. H6 as sister taxon to other placozoans in the genus *Cladtertia*. The given topology was fully supported (1.0 bayesian probability) by Bayesian inferences of all three data matrices: concatenated nucleotide and amino acid sequences of protein‐coding genes and concatenated ribosomal DNA sequences. Bootstrap support for Maximum Likelihood analyses of the three data matrices is indicated beside the notes. The small image captures highlight the striking morphological differences between the typical amoeboid (roundish) shape in the class Uniplacotoma (exemplified here with images from *Trichoplax adhaerens* and *Hoilungia hongkongensis* [upper and lower two images, respectively; Eitel et al., 2018]) and the ramified habitus of *Polyplacotoma mediterranea* (photograph by H.J. Osigus, 2020; WoRMS) belonging to the class Polyplacotomia. Note that some haplotypes are missing in this phylogram due to the lack of complete mitochondrial genome sequences. aa, amino acid; CDS, coding sequence; rDNA, ribosomal DNA.

We obtained placozoan mitochondrial sequence data from Rhodopidae samples (Sevigny et al., 2021) by using all available sequencing reads, including mitochondrial‐enriched and non‐enriched metagenomic data. The combination of these datasets improved the coverage and sequence quality of the final assembled placozoan mitogenome contigs. However, the vast majority of placozoan reads in the snail genomic DNA stems from the enriched datasets for all identified placozoa (Table 1), indicating that the mitochondrial DNA enrichment by Sevigny and co‐workers (unintendedly) also worked for placozoans. The metagenome assemblies were screened for placozoan mitochondrial sequences, resulting in the identification of four haplotypes in the *Helminthope* sp. sea slug metagenome assembly, including two previously known (H3 and *Cladtertia* H6) and two newly discovered haplotypes, *Hoilungia* H26 and H27 (clade VII). Additionally, a new haplotype, H28 (clade VII), was identified in the *Rhodope* sp. sea slug metagenome assembly. The haplotypes were assigned to previously established placozoan molecular clades or formally described genera based on phylogenetic inferences (Figure 1) using concatenated amino acid sequences of the 12 encoded mitochondrial genes (COX1‐3, COB, ATP6, NAD1‐6, and NAD4L) and additionally by comparison of the NAD3 protein sequence (see below).

**TABLE 1:** Completeness of placozoan mitochondrial genomes isolated from two Rhodopidae sea slug specimens.

Placozoan lineage	Source	mt chromosome^a^	mt CDS^a^	mt rDNA^a^	Mean coverage of mt chromosome/mt contigs: Enriched	Mean coverage of mt chromosome/mt contigs: Non‐enriched
H3	*Helminthope* sp.	~61.9%	58.3%	78.2%	43.8x	0.2x
*Cladtertia* H6	*Helminthope* sp.	~99.9%	100%	100%	809.5x	3.8x
*Hoilungia* H26	*Helminthope* sp.	~99.9%	100%	100%	392.5x	1.0x
H27	*Helminthope* sp.	~98.2%	100%	100%	621.5x	2.9x
H28	*Rhodope* sp.	~80.6%	95.1%	97.8%	71.6x	0.5x

^a^ Approximation based on alignment with the complete mitochondrial genome sequence of the next closest related species according to Figure 1.

**FIGURE 2:**
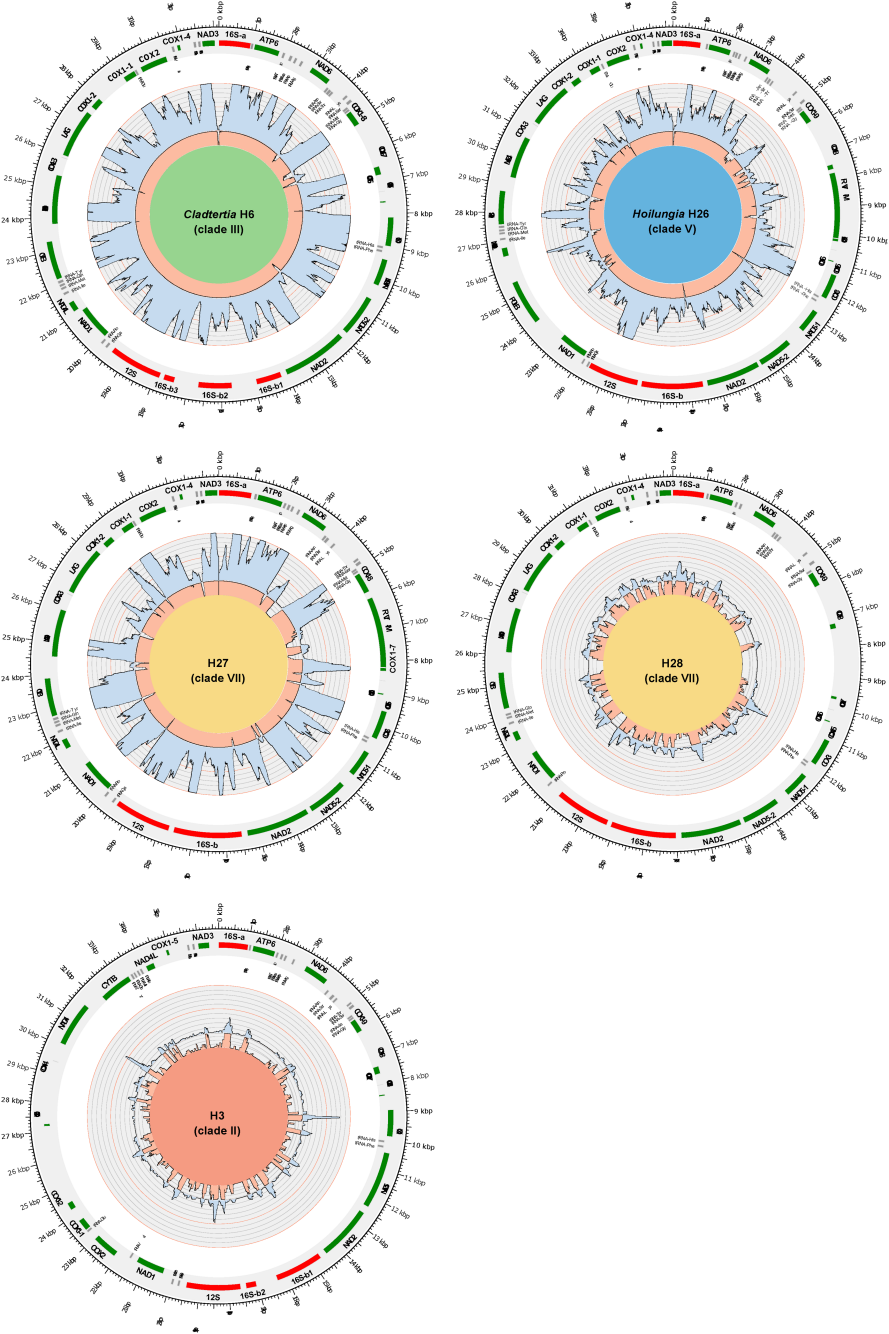
Placozoan mitochondrial genome architecture isolated from two Rhodopidae sea slug specimens. Shown are plots of the five isolated partial or almost complete placozoan mitochondrial genomes isolated from the mollusc species *Helminthope* sp. (*Cladtertia* H6, *Hoilungia* H26, and the haplotypes H3 and H27) and *Rhodophe* sp. (haplotype H28). The outer circle indicates protein‐coding genes as green blocks, ribosomal DNAs in red, and tRNAs in gray, respectively. Note that COX1 (1–9), NAD5 (1–2), and 16S (a and b) are split into multiple exons in most cases, as described previously. The direction of coding sequences and linkage of exons are not shown (see Miyazawa et al., 2021) for details on placozoan mitochondrial genome structures). The coverage by short metagenomic reads is shown in light blue. It was capped at 1000×. Each gray subcircle represents 100× and each red 500× coverage, respectively. The light red coverage plot shows a magnification of the coverage on the lower end (from 0–100×). Gaps in the mitochondrial genome are indicated by black blocks (innermost circle). The length of gaps in *Hoilungia* H26 and H27 is based on the alignment of the mitochondrial genome sequences with the closely related *Hoilungia* H4 and H24, respectively. The published H24 sequence was used as a reference to map the H28 contigs since it is the closest related haplotype sequenced to date. The length of the gap region is, therefore, based on the H24 sequence and the actual length, as well as the mitochondrial structure, might slightly differ in H28. To map the H3 contigs, the published mitochondrial genome sequence of this haplotype was used. The background color of the plots matches the color code used for clades in the phylogenetic tree in Figure 1. LAG, Open Reading Frame (ORF) showing high similarities to a group I intron‐encoded LAGLIDADG endonuclease domain; POLB, ORF showing similarity with a fungal DNA‐directed DNA polymerase type B; RVT‐IM, ORF containing a reverse transcriptase domain and a group II intron maturase domain.

Confirmation of the accuracy of assembled placozoan mitochondrial scaffolds/contigs was achieved through back‐mapping of trimmed reads. The combination of a read length of 250 bp, insert size ranging from 400 to 800 bp, and genetic distances, along with medium to high sequence coverage (as shown in Table 1), provided reliable discrimination of the five identified placozoan haplotypes. Upon mapping uncorrected reads back to the scaffolds/contigs (not shown), it was observed that the intrinsic read error correction (performed prior to assembly) was not compromised by the presence of multiple placozoan haplotypes or mollusc reads. Notably, despite the high number of identified placozoan genera in the *Helminthope* sp. sea slug metagenomic data, the high‐quality and long (250 bp) Illumina paired‐end reads provide enough support to differentiate between these, enabling the correct (partial) mitogenome assembly of each. Based on alignments of the identified placozoan mitochondrial scaffolds/contigs to the most closely related complete publicly available mitochondrial chromosome, the completeness of the five identified placozoan haplotypes ranged from 61.9% to 99.9% (see Table 1 and read coverage in Figure 2), indicating that enrichment for gastropod mitochondrial sequences also resulted in high enrichement for placozoan mitochondrial sequences. Other samples from the original mitochondrial enrichment study (Sevigny et al., 2021), including the phyla Annelida, Chordata, Echinodermata, Nemertea, and additional mollusc species, were screened, but not a single unambiguously assignable placozoan sequence was found in any of these taxa, highlighting the special predator–prey relationship of rhodopid sea slugs and placozoans.

To broaden our understanding of the potential prey consumed by the rhodopid sea slug species under investigation, we conducted screenings for additional mitochondrial and ribosomal DNA signatures. Our BLAST searches revealed significant identity matches to certain nemertean taxa of the interstitial genus *Ototyphlonemertes* Diesing, 1863 (von Döhren & Bartolomaeus, 2020) in both sea slug metagenomic datasets, along with hits to ribosomal sequences from annelids, molluscs, and flatworms (Table S1). However, the vast majority of hits could be assigned to placozoans, as outlined above.

### 3.2 Comparative placozoan mitogenomics

Within the *Cladtertia* genus (formerly Clade III), the newly assembled mitochondrial genomes of *Cladtertia* H6 showed identical structure to previously published mitochondrial genomes of *Cladtertia* H8 and *C. collaboinventa* (H23; Miyazawa et al., 2021; Signorovitch et al., 2007). This implies the position and length of the eight cox1 (including the cox1 micro exon; Osigus et al., 2017), two nad5, and four 16S exons (split among two 16S parts), respectively. All four haplotypes of *Cladtertia* had highly conserved mitochondrial genome sizes between 32,154 and 32,980 bp, with only a small gap of ~54 bp present in the *Cladtertia* H6 mt genome. The sequence identities of the 12 concatenated protein coding sequences between the four *Cladtertia* lineages were high, ranging from 95.77% to 98.88% on the nucleotide level and from 95.69% to 98.63% on the amino acid level, respectively. A structural synapomorphy was identified in *Cladtertia* H8/*C. collaboinventa* (H23) with an inversion of the region spanning tRNA‐Thr and tRNA‐Lys. Apart from this inversion, the mitochondrial genome architecture was identical in all four *Cladtertia* haplotypes, including preserved gene orders and intron numbers and positions.

The newly discovered mitochondrial haplotype H26 in the *Hoilungia* genus (formerly clade V) showed high similarity with other members of the genus, with nucleotide CDS percentage identities ranging from 99.03% to 99.96% and amino acid identities ranging from 99.13% to 99.93%. At the complete mitochondrial genome level, H26 was almost identical to H4, with a 99.66% nucleotide identity. The mitochondrial genome architecture of H26 was also identical to H4, including the gene order, numbers, and positions of introns. Additionally, the unique position of the DNA‐directed DNA polymerase type B gene, which is an autapomorphy of the *Hoilungia* within the Placozoa (Miyazawa et al., 2021; Signorovitch et al., 2007), was confirmed in H26 and H4. This position is located between NAD1 and NAD4L in H4 and H26, while it is found between tRNA‐Lys and tRNA‐Thr in H9, H13, and H15, likely due to a translocation and inversion event. The expanded placozoan dataset also confirms the exclusive presence of the polymerase B gene in the *Hoilungia* genus.

The newly discovered H28 in clade VII (no genus defined yet) is closely related to H24 and H27. Sequence identity values within this clade ranged between 91.17% and 97.78% on the nucleotide level and 90.23% and 97.83% on the amino acid level, respectively. H27 shows an inversion between the two copies of tRNA‐Ser, which is not present in H28. However, the mitochondrial genome architecture of all three haplotypes in clade VII is otherwise identical, including gene order and intron numbers and positions.

### 3.3 Comparative placozoan mitochondrial gene sequence divergence

**FIGURE 3:**
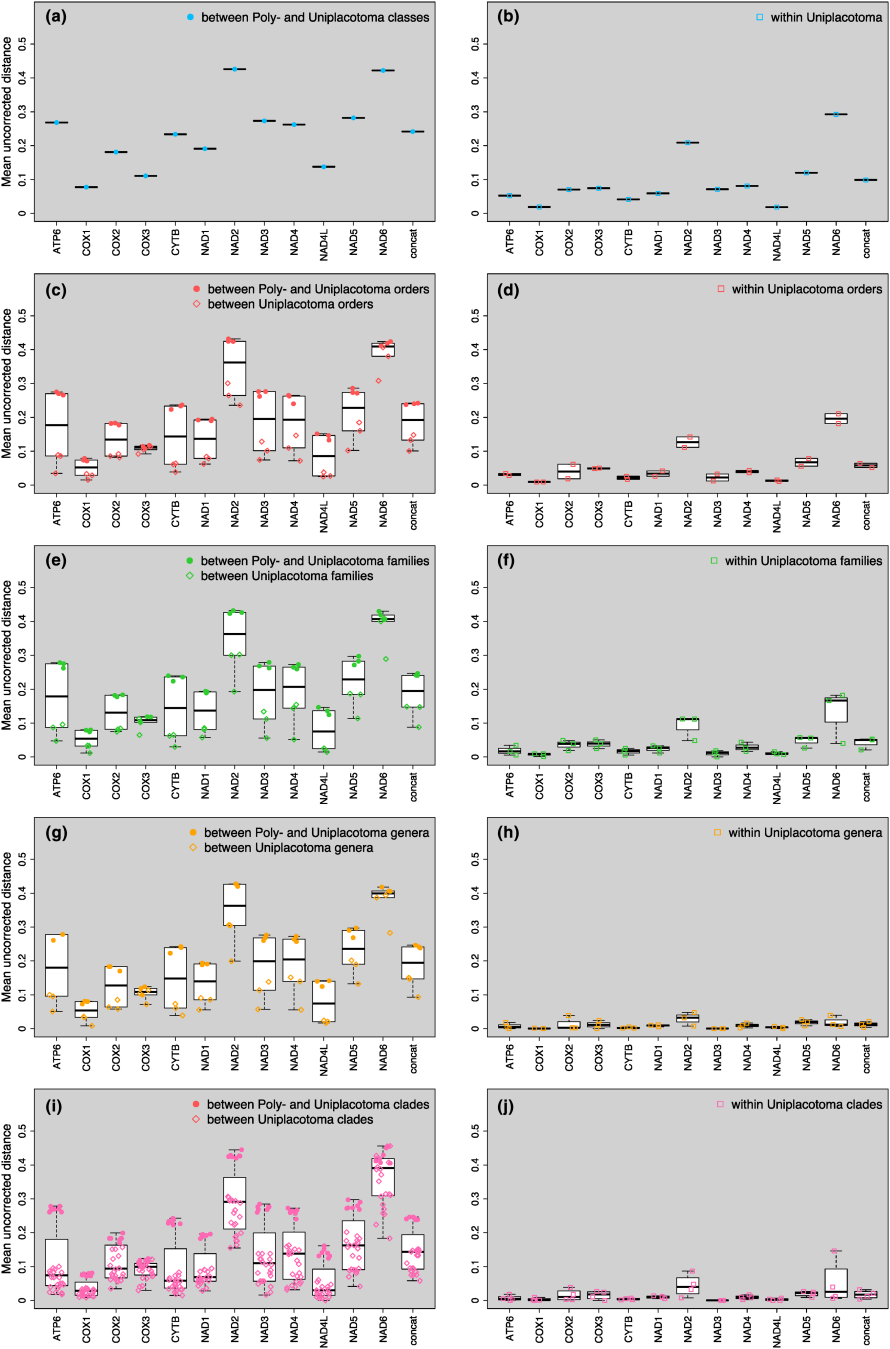
Calculated uncorrected pairwise genetic distances of placozoan mitochondrial proteins. Shown are the uncorrected pairwise distances for the 12 commonly encoded proteins of placozoan mitochondrial genomes (separately as well as concatenated) for orders within classes (a, b), families within orders (c, d), genera within families (e, f), species within genera (g, h), and haplotypes within clades (i, j). In the left panels, the inter‐group distances are detailed for the two placozoan classes, while on the right panels, the intra‐group distances of placozoans only belonging to the class Uniplacotoma are presented, respectively. As expected, distances between the classes are larger than within Uniplacotoma. Note that in the class Polyplacotomia, only one placozoan representative has been described to date, which hinders intra‐group comparisons. concat, 12 concatenated mitochondrial proteins.

In order to assess differences between mitochondrial and nuclear datasets in relation to the established placozoan phylogeny (Tessler et al., 2022), we conducted pairwise mean uncorrected distance calculations at various phylogenetic levels and taxonomic ranks (Figure 3) for both amino acid sequence alignments of individual mitochondrial proteins and the 12 concatenated alignments. Comparing sequence divergence within and between the two placozoan classes Uni‐ and Polyplacotomia, we observed significant differences. Genetic distances are higher between the classes but comparatively lower within more diverse Uniplacotomia, decreasing with lower taxonomic ranks as would be expected in a functional taxonomic system (i.e., more recently separated species show less genetic variation; see Figure S1 in Supplemental Information). Furthermore, interspecific differences are higher between various taxonomic ranks in Uniplacotomia than within them (compare the left and right panels in Figure 3). The between‐group distances in NAD3 are of particular interest, revealing consistent patterns across genera and clades, respectively. On one hand, these distances delineate the two placozoan classes and various depths within different placozoan families. On the other hand, NAD3 sequences exhibit high levels of conservation both within genera and clades, as illustrated in Figure S2 (Supplemental Information).

## 4 DISCUSSION

### 4.1 Taxonomic implications

The phylum Placozoa was for a long time believed to comprise only one species, *Trichoplax adhaerens* (Schulze, 1883, 1891), despite ecological variations. A second species was described but not rediscovered (Monticelli, 1893). After one century with limited research on the Placozoa, molecular genotyping revealed high genetic diversity (Voigt et al., 2004), as confirmed by subsequent studies (Eitel et al., 2013; Eitel & Schierwater, 2010; Miyazawa & Nakano, 2018; Signorovitch et al., 2006). So far, 26 genetic lineages have been identified in tropical, subtropical, and temperate waters, grouped into seven molecular clades. New species and higher order taxonomic ranks are anticipated based on genetic (Eitel et al., 2018; Tessler et al., 2022; Voigt et al., 2004) and morphological data (Guidi et al., 2011; Romanova et al., 2021).

Our analysis of metagenome datasets from Rhodopidae sea slug specimens that feed on placozoans discovered five different placozoan haplotypes, including three new ones, two of which group within clade VII. We obtained the nearly complete Cladtertia H6 mitochondrion sequence, for which so far only sequence fragments have been known. The inclusion of this new mitogenomic data supports recent findings on the placozoan phylogeny. Our genetic distance analysis confirmed the taxonomic assignment of placozoan haplotypes based on comparative genomics and phylogenomics of whole nuclear genome data (Tessler et al., 2022). The decreasing within‐group genetic distances from classes to genera strongly support the proposed Linnean systematic framework of Placozoa.

Our genetic distance analysis incorporating data from 18 placozoan mitochondrial genomes revealed diagnostic differences in the NAD3 protein sequence at the inter‐generic and inter‐clade levels in placozoans, while sequences *within* genera and clades are identical. This comprehensive analysis allows, for the first time, the use of NAD3 as a diagnostic marker to assign placozoan samples to existing or new clades and genera. For future definition of genera in Placozoa, we propose replacing the rather ambiguous 16S rDNA fragment with the diagnostic NAD3 protein sequences. However, we still recommend using 16S rDNA for identifying new haplotypes at subgeneric levels. The preservation of type specimens for clades with so far unconfirmed taxonomic assignments to a genus will be the critical step to entirely replacing the current placozoan clade system by a genuine Linnaean taxonomic system, as previously envisioned (Eitel et al., 2018; Tessler et al., 2022).

### 4.2 Ecological and biological implications

The improved understanding of placozoan molecular biodiversity and taxonomy sheds light on their ecology and biology. The discovery of the placozoan haplotype H3 from seafloor sediment in an eastern Pacific location challenges its assumed restricted occurrence in the Caribbean (Eitel et al., 2013), indicating wider distribution and adaptability. Based on the new finding, we hypothesize that haplotype H3 might also be present in other places with similar habitats, particularly when sediment sampling is extended.

Placozoan endemism, particularly in the class Uniplacotomia, appears increasingly less likely due to extensive molecular data from various sampling sites (Eitel et al., 2013; Eitel & Schierwater, 2010; Miyazawa & Nakano, 2018; Pearse & Voigt, 2010; Signorovitch et al., 2006; Voigt et al., 2004). However, accurately assessing placozoan population structure remains challenging despite sampling advancements. High‐throughput environmental DNA sequencing methods hold promise for better understanding placozoan biodiversity, phylogeography, and population dynamics.

Placozoans are typically grazers on solid surfaces like coral reefs, rocks, and mangrove roots (Eitel et al., 2013; Eitel & Schierwater, 2010; Signorovitch et al., 2006; Voigt et al., 2004). Their presence inside seafloor sediment has never been documented before. The presence of Placozoa in the gut contents of a *Helminthope* sp. sea slug, particularly considering that at least one congeneric sea slug species is a fully interstitial predator in deeper regions of the sediment, implies a potentially broader ecological adaptability for placozoans. While sea slugs of the genus *Rhodope* are also recorded to live on solid surfaces outside of the sediment interstitial (e.g. on the sediment surface and on algae‐covered rocks), *Helminthope* slugs are known solely from the interstitial (Jörger et al., 2020; Wilson et al., 2017). Extraction of *Helminthope* from sediment requires special methods, and the highly derived worm‐like morphology (Brenzinger et al., 2013; Salvini‐Plawen, 1991) is strongly consistent with an entirely interstitial lifestyle, as is corroborated by our analysis of other taxa in the molecular data (putatively prey), particularly interstitial nemerteans. This, in sum, strongly indicates an interstitial lifestyle of *Helminthope* spp. and, consequently, the presence of placozoans in the interstitium.

Previously known to inhabit benthic habitats and the water column (Eitel et al., 2013; Eitel & Schierwater, 2010; Pearse & Voigt, 2010), placozoans have now been found in seafloor sediments, expanding their ecological niche range. The seafloor interstitium, a large marine habitat, may play a crucial role in their sexual reproduction and the development of unknown life cycle stages. Interestingly, placozoans do not show adverse reactions when consumed by sea slugs (Cuervo‐González, 2017), and being preyed upon may even benefit them in completing their unknown life cycle. While the exact life cycle stage of placozoans taken up by Rhodopidae sea slugs remains elusive, it is possible that the sea slug digestive gland supports the completion of the placozoan life cycle under specific conditions yet to be identified. Furthermore, preying on placozoans could also support their dominant mode of asexual reproduction, binary fission, by fragmenting individuals, followed by regrowth of the divided parts.

The abundance of placozoan genomic reads in the Rhodopidae datasets confirms their specific predation on placozoans, supporting previous observations (Cuervo‐González, 2017; Riedl, 1959). Undigested DNA from various placozoan genera provides new insights into the composition of the placozoan community in an understudied habitat. The unprecedented scale of local diversity observed in this study surpasses previous findings of sympatric clades (Signorovitch et al., 2006). While the mobility of Rhodopidae sea slugs in the interstitial habitat is not well understood, their small size and limited individual range suggest that the analyzed molecular data likely represent placozoans from multiple genera within a small area and consumed over a short period. The limited sampling size indicates that the actual diversity of placozoans may be much higher than previously estimated (Eitel et al., 2013), potentially encompassing hundreds of unidentified taxa living sympatrically in marine habitats. The interstitial pore space, a protected habitat suitable for various small organisms (e.g., Schmidt‐Rhaesa, 2020), including placozoans, may serve as an underappreciated environment for certain placozoan life stages.

The discovery of placozoans in the interstitium allows raising questions about their origin. The placozoan species *Polyplacotoma mediterranea*, despite being found on hard substrates, displays a branching pattern with an expanded surface, suggesting it may have adapted to primarily inhabit sediment‐filled spaces and consume limited organic matter. Its phylogenetic position as a sister to round‐shaped placozoans (Tessler et al., 2022) supports the hypothesis that uniplacotomian placozoans reduced their habitus complexity as they adapted to a surface‐grazing lifestyle. They might have extended their habitat range to include larger materials like benthic stones and corals in shallow coastal regions with abundant biofilms. To survive in such environments with high wave activity, they may have undergone morphological simplification to reduce their surface area while increasing mobility and dispersal. Confirming this hypothesis requires a comprehensive understanding of placozoan diversity and life history through extensive coastal sediment sampling and molecular analyses.

The exploration of metagenomic DNA sequences holds significant potential for advancing biodiversity, fundamental biology, and ecological studies. Within biodiversity research, a crucial distinction is between discovery and later description, especially when delving into metagenomic DNA sequences of understudied groups, of which prime examples are elusive meiofaunal groups or diverse ones such as copepods. Bridging the biodiversity discovery gap (Bouchet et al., 2023; Hortal et al., 2015) and describing the unnamed species identified particularly for these require extended collection efforts in previously undersampled areas and habitats, followed by systematic, taxonomic, and molecular genetic approaches. By now, a treasure trove for insights into molecular diversity and species interactions also lies in publicly available metagenomic genetic material.

In our present analyses of data derived from the gut contents of predators inhabiting seafloor interstitial spaces, we have discovered five placozoan genetic lineages (three of which have not been known before) belonging to four described placozoan genera. Taken together, this enhances our comprehension of placozoan diversity, thereby enriching our understanding of the group's distribution and ecology, and elucidates previously unknown predator–prey interactions in one of the largest marine benthic habitats, the seafloor interstitium. This approach mirrors the discoveries made, e.g., in deep‐sea mollusc research (Bergmeier et al., 2021), illustrating its promise in unveiling hidden facets of marine ecosystems.

## AUTHOR CONTRIBUTIONS

**Michael Eitel:** Conceptualization (lead); data curation (lead); formal analysis (lead); investigation (lead); methodology (lead); project administration (lead); resources (equal); validation (lead); visualization (lead); writing – original draft (lead); writing – review and editing (lead). **Hans‐Jürgen Osigus:** Conceptualization (equal); formal analysis (equal); investigation (equal); methodology (equal); writing – review and editing (equal). **Bastian Brenzinger:** Investigation (equal); writing – review and editing (equal). **Gert Wörheide:** Formal analysis (equal); funding acquisition (lead); project administration (equal); resources (lead); software (lead); supervision (lead); validation (equal); writing – review and editing (equal).

## CONFLICT OF INTEREST STATEMENT

The authors declare no conflict of interest.

## Supporting information


Figures S1.‐S2.

Table S1.

## Data Availability

Our results were based on public metagenomic DNA datasets (Sevigny et al., 2021): GenBank BioProject accession PRJNA483473 (BioSample ID SAMN14489971 and SAMN14489968) with associated NCBI SRA accessions SRR12246750, SRR12246747, SRR12246802, and SRR12246798. Newly generated mitochondrial genome assemblies are available in the European Nucleotide Archive (https://www.ebi.ac.uk/ena/browser/home) under accessions ERZ23789290, ERZ23789291, ERZ23789292, and ERZ23789302. Scripts for assemblies and data analyses as well as associated data (including annotated mitochondrial genomes) are deposited in a GitHub repository (https://github.com/PalMuc/beauty_in_the_beast), v1.0.0 (25‐07‐2023), archived at Zenodo (https://doi.org/10.5281/zenodo.8181901).
