# Supplementary material for: Beauty in the beast – Placozoan biodiversity explored through molluscan predator genomics
Source: Ecol Evol. 2024 Apr 11;14(4):e11220. doi: 10.1002/ece3.11220 (PMC11007570; doi:10.1002/ece3.11220)
Supplement: Supplementary file 1 — Figures S1.‐S2. [file ECE3-14-e11220-s001.docx]

**Supplemental Information for:**

**Beauty in the beast - Placozoan biodiversity explored through molluscan predator genomics**

Michael Eitel, Hans-Jürgen Osigus, Bastian Brenzinger, Gert Wörheide

**Table of Contents:**

| **Figure S1** | Page 2 |
| --- | --- |
| **Figure S2** | Page 3 |

**Figure S1. Genetic distances of mitochondrial genes confirm the established taxonomic system of the Placozoa.**

Calculated uncorrected pairwise genetic distances for twelve individuals plus all combined (concat) mitochondrial genes on the amino acid level. Each data point represents the average of all mean group distances between or within orders within classes (‘class’), families within orders (‘order’), genera within families (‘family’), and species within genera (‘genus’). Genetic distances are decreasing with lower order taxonomic ranks.

**Figure S2. Using NAD3 as a marker to define genera in the Placozoa.**

The complete sequence alignment of mitochondrial NAD3 is depicted here, with amino acid substitutions highlighted in distinct colors (a). The amino acid sequences within phylogenetic clades and within the described genera *Trichoplax*, *Hoilungia* and *Cladtertia* exhibit 100% identity, respectively. This pattern is also evident when examining mean uncorrected pairwise distances (b). Both intra-clade and intra-generic distances are zero (left bars). Moreover, comparing distances ‘between genera’ to ‘between clades’ reveals similar distribution of distances (right bars): two groups, one with higher and one with lower distances. Collectively, data from 18 placozoans advocate for the utilization of the NAD3 protein sequence to classify placozoans into existing genera and to delineate new ones.
