# Supplementary material for: Beauty in the beast – Placozoan biodiversity explored through molluscan predator genomics
Source: Ecol Evol. 2024 Apr 11;14(4):e11220. doi: 10.1002/ece3.11220 (PMC11007570; doi:10.1002/ece3.11220)
Supplement: Supplementary file 2 — Table S1. [file ECE3-14-e11220-s002.zip › Table s1 caption.docx]

Table S1. Summary of contigs with highest similarities to sea slug host, placozoan and nemertean mitochondrial and ribosomal DNA sequences, respectively.
